# Hepatitis B virus pre-S deletion mutations are a risk factor for hepatocellular carcinoma: a matched nested case–control study

**DOI:** 10.1099/vir.0.2008/002824-0

**Published:** 2008-11

**Authors:** Zhong-Liao Fang, Caroline A. Sabin, Bai-Qing Dong, Shao-Chao Wei, Qin-Yan Chen, Kong-Xiong Fang, Jin-Ye Yang, Jian Huang, Xue-Yan Wang, Tim J. Harrison

**Affiliations:** 1 Department of Medicine, UCL Medical School, London W1T 4JF, UK; 2 Guangxi Zhuang Autonomous Region Center for Disease Prevention and Control, Jin Zhou Road, Nanning, Guangxi 530028, PR China; 3 Research Department of Infection and Population Health, Division of Population Health, UCL Medical School, University College London, London NW3 2PF, UK; 4 Sanitary and Antiepidemic Station of Long An, ChengXi Road, Cheng Xiang Town, Long An, Guangxi 532700, PR China

## Abstract

A matched nested case–control study of 33 paired cases and controls was conducted, based on a study cohort in Long An county, Guangxi, China, to determine whether infection with hepatitis B virus (HBV) with pre-S deletions is independently associated with the development of hepatocellular carcinoma (HCC), without the confounding effects of basal core promoter (BCP) double mutations. The prevalence of pre-S deletions was significantly higher in HCC (45.5 %, 15 of 33) than the controls (18.2 %, 6 of 33) (*P*<0.01), under the control of the influence of BCP double mutations. Most of the pre-S deletions occurred in, or involved, the 5′ half of the pre-S2 region and the difference between HCC (93.3 %, 14 of 15) and controls (66.7 %, four of six) was significant for this region (*P*=0.015). There was no significant difference in pre-S deletions between the BCP mutant group and BCP wild-type group (*P*>0.05), nor was the prevalence of pre-S deletions significantly different between genotypes B and C (*P*>0.1). These results suggest that pre-S deletions constitute an independent risk factor for HCC and their emergence and effect are independent of BCP mutations. The 5′ terminus of pre-S2 is the favoured site for the deletion mutations, especially in HCC cases. Further prospective studies are required to confirm the role of these mutations in the development of HCC.

## INTRODUCTION

Chronic hepatitis B virus (HBV) infection is the most important aetiology of hepatocellular carcinoma (HCC) in Asia (Beasley *et al.*, 1981). However, the mechanisms of oncogenesis are obscure. Recently, viral factors associated with the development of HCC have become a major focus for research. The common precore mutation (G_1896_A) and mutations in enhancer II (C_1653_T) and the basal core promoter (T_1753_V and the double mutations A_1762_T and G_1764_A) have been reported to be associated with the development of HCC (Liu *et al.*, 2006; Tanaka *et al.*, 2006; Chen *et al.*, 2006a; Yuen *et al.*, 2008). Perhaps the most convincing association is with virus with double mutations in the basal core promoter (BCP) (Hsia *et al.*, 1996; Fang *et al.*, 1998, 2002; Baptista *et al.*, 1999; Kao *et al.*, 2003). A recent prospective study of a cohort of 2258 hepatitis B surface antigen (HBsAg)-positive individuals in Long An county, Guangxi, China showed that BCP double mutations are an aetiological factor of HCC (Fang *et al.*, 2008). Mutations in the BCP may also result in amino acid substitutions in the X protein and the A_1762_T, G_1764_A mutations result in two, L_130_M and V_131_I; however, these changes decrease the ability of the protein to transactivate transcription, at least as far as expression of the viral precore and pregenomic RNAs are concerned (Li *et al.*, 1999).

HBV can be divided into eight genotypes (designated by capital letters A to H) based on an intergroup divergence of 8 % or more in the complete nucleotide sequence (Okamoto *et al.*, 1988; Arauz-Ruiz *et al.*, 2002) and these display remarkable geographical variation. Genotypes B and C are predominant in Asia (Yu *et al.*, 2005) and have been reported to have clinical relevance (Kao, 2002). However, the precise role of these two genotypes in the development of HCC remains controversial (Chan *et al.*, 2004; Yu *et al.*, 2005; Sumi *et al.*, 2003; Yuen *et al.*, 2008). The association between HBV genotype C and HCC may not be attributable to genotype per se but rather to the high prevalence of BCP double mutations in patients with genotype C (Yuen *et al.*, 2004).

The emergence of persistently infected individuals of HBV with deletions in the pre-S region has been recognized for many years (Santantonio *et al.*, 1992) and the mutations have been reported to be more common in genotypes B and C than in other HBV genotypes (Huy *et al.*, 2003). Although there is increasing evidence of association of these mutations with severe liver disease (Tai *et al.*, 2002; Huy *et al.*, 2003), their clinical significance is rather obscure, especially their association with HCC. A recent study from Taiwan reported that the combination of pre-S deletion mutations and BCP double mutations, rather than either alone, was associated with the development of HCC (Chen *et al.*, 2006a). Several subsequent studies also reported that pre-S deletions are associated with the development of HCC. However, this association is not convincing without exclusion of the confounding effect of BCP double mutations (Choi *et al.*, 2007; Lin *et al.*, 2007; Gao *et al.*, 2007). The occurrence of pre-S deletions and BCP mutations is associated with HBV genotype (Sugauchi *et al.*, 2003) and both are of higher prevalence in genotype C than in other genotypes (Kao *et al.*, 2003; Sugauchi *et al.*, 2003). It is possible that the association between pre-S deletions and HCC may be not attributable to pre-S deletions per se but rather to the high prevalence of BCP double mutations in genotype C.

The aim of this study was to determine whether the association of pre-S deletions with the development of HCC is independent of BCP double mutations. In this matched nested case–control study, both cases and controls were selected from the Long An cohort, Guangxi, China (Fang *et al.*, 2008).

## METHODS

### The Long An cohort.

In order to determine the value of screening carriers of HBsAg for virus with core promoter double mutations as a marker of extremely high risk of developing HCC, a cohort of 2258 hHBsAg-positive subjects, 30–55 years of age, was recruited in Guangxi, China (Fang *et al.*, 2008). Informed consent in writing was obtained from each individual. The study protocol conforms to the ethical guidelines of the 1975 Helsinki Declaration and has been approved by the Guangxi Institutional Review Board and the UCL Committee on the Ethics of Non-NHS Human Research (Project number 0042/001).

Our Chinese study team comprises doctors from Centers for Disease Prevention and Control (CPDC) of Long An county and the CPDC of Guangxi Province. From 1 March 2004, the study teams travelled to 128 villages in each of the 12 townships of Long An county to visit agricultural workers aged 30–55 to collect a 3 ml sample of blood by venepuncture for screening for HBsAg. All samples were tested for HBsAg and positive samples were tested in China for HBV DNA by using nested PCR. We also detected and excluded those samples positive for anti-hepatitis C virus (HCV) to eliminate the confounding effect of HCV infection on the incidence of HCC. We started to follow up the study subjects from 1 July 2004. Each study subject completed a one-page questionnaire at the first visit and provided a serum sample every six months for the assessment of virological parameters and alpha fetoprotein (AFP) concentrations and was monitored for HCC by ultrasonography (US). All cases of HCC diagnosed were confirmed at the Medical University of Guangxi, the Cancer Institute of Guangxi or the Hospital of Guangxi (Nanning) using criteria set by the Chinese Anti-Cancer Association.

### HCC cases and controls.

After 36 months follow-up, 61 individuals were diagnosed with HCC and sufficient volumes of serum remained from 33 (25 males and 8 females, 29 of whom were infected with HBV with BCP double mutations) for this study. A control was selected from the cohort for each case, matched for age (where possible, within 12 months), sex and the status of BCP sequence (wild type or double mutation) (Table 1).

### Serological testing.

Sera were tested for HBsAg, HBeAg/anti-HBe, anti-HCV antibodies and AFP using enzyme immunoassays (Zhong Shan Biological Technology Company). Alanine aminotransferase (ALT) levels were determined using a Reitman kit (Sichuan Mike Scientific Technology Company).

### Nested PCR for HBV DNA and nucleotide sequencing.

DNA was extracted from 85 μl serum by Pronase digestion followed by phenol/chloroform extraction. For nested PCR, first round PCR was carried out in a 50 μl reaction using primers LSOB1 (nt 2739–2762, 5′-GGCATTATTTGCATACCCTTTGG-3′) and MDN5R (nt 1794–1774, 5′-ATTTATGCCTACAGCCTCCT-3′), or P2 (Gunther *et al.*, 1995), with 5 min hot start followed by 30 cycles of 94 °C for 30 s, 50 °C for 30 s and 72 °C for 90 s. A second round of PCR was carried out on 5 μl of the first round products in a 50 μl reaction using primers LSBI1 (nt 2809–2829, 5′-TTGTGGGTCACCATATTCTT-3′) and XSEQ1R (nt 1547–1569, 5′-CAGATGAGAAGGCACAGACGGGG-3′) and the same amplification protocol as the first round.

Products from the second round were confirmed by agarose gel electrophoresis and then purified using the GenElute PCR Clean-up kit (Sigma) according to the manufacturer's instructions. Cycle sequencing was carried out directly on both strands using 2 μl purified amplicon DNA and primer LSBI1 or ADELN (nt 432–453, 5′-TAGTCCAGAAGAACCAACAAG-3′) and a BigDye Terminator V3.1 Cycle Sequencing kit (Applied Biosystems) according to the manufacturer's instructions. The nucleotide sequences derived in this study have been submitted to GenBank/EMBL/DDBJ under accession numbers FM211353–FM211418.

### HBV genotyping.

HBV genotyping for both cases and controls were determined using the sequences above and the STAR program [http://www.vgb.ucl.ac.uk/starn.shtml (Myers *et al.*, 2006)] and the NCBI Genotyping Tool (http://www.ncbi.nlm.nih.gov/projects/genotyping/formpage.cgi).

### Statistical analysis.

The statistical comparisons were performed using Pearson's *χ*^2^ tests, McNemar's test and Fisher's exact test. All *P*-values were two-tailed and *P*<0.05 was considered to be significant. Univariate and multivariate conditional logistic regression analyses were performed using the Statistical Package of Statistical Analysis System (SAS version 9.0). Variables with *P*<0.05 on univariate analysis were analysed by stepwise multivariate analysis for independent risk factors associated with HCC development. All *P*-values were two-tailed and *P*<0.1 was considered to be significant.

## RESULTS

### The association of pre-S deletions and HCC

Pre-S deletions were found in HBV DNA from 15 of 33 (45.5 %) HCC cases tested (Table 2). In contrast, deletions were found in only six of 33 (18.2 %) controls (*P*<0.01). There was no significant difference in the prevalence of deletions between males and females (*P*>0.1). On univariate analysis, pre-S deletion was independently associated with the development of HCC but HBeAg, anti-HBe, ALT concentrations and genotypes were not. On multivariate analysis, pre-S deletion remained independently associated with the development of HCC (hazard ratio=7, 95 % confidence limits=0.861–56.894) (Table 3). Because the BCP sequences of matched cases and controls are the same, the results suggest that the association of pre-S deletions with the development of HCC is independent of BCP double mutations.

**Fig. 1. f1:**
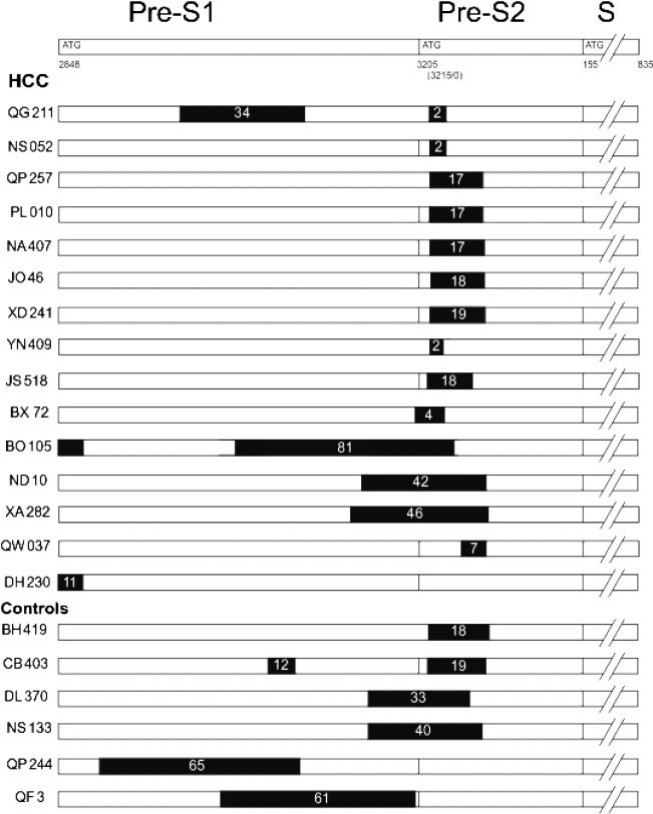
Location of deletions detected in HBV from HCC cases and controls. The upper bar depicts the wild-type surface ORF; vertical lines indicate the initiation (pre-S1, pre-S2 and S) and termination codons (nucleotide positions are according to standard nomenclature). The remaining bars represent the surface ORFs of HBV from 15 HCC cases and six controls; black rectangles indicate deleted sequences and the numbers of codons deleted. The pre-S2 initiation codon is absent from some isolates.

The locations of the deletions are shown in Fig. 1. All of the deletions are in-frame so that the integrity of the polymerase ORF is maintained. Among the 15 HBV deletion mutations in the HCC group, one (6.7 %) occurred in pre-S1, nine (60 %) in the 5′ half of the pre-S2 region and four (26.7 %) cases had mutations that removed the pre-S2 initiation codon and adjacent sequences. Two of the fifteen had two deletions (one in pre-S1 and another in the 5′ half of the pre-S2 region). In total, 93.3 % of pre-S deletions in this group occurred in or involved the 5′ terminus of the pre-S2 region. In contrast, of the HBV deletion mutations in the control groups, 33.3 % (2/6) occurred in the pre-S1 region, 16.7 % (1/6) in the pre-S2 region and 50 % (3/6) had mutations that removed the pre-S2 initiation codon and adjacent sequences. In total, 66.7 % (4/6) of the pre-S deletions in the control group occurred in or involved the 5′ terminus of pre-S2. The prevalence of deletions occurring in or involving the pre-S2 region is higher in HCC than in the controls (*P*=0.015).

Expression of the middle surface protein also may be abrogated by point mutations in the pre-S2 initiation codon. That codon was changed in 18.2 % (6/33) of HCC cases but only 12.1 % (4/33) of controls, although the difference is not statistically significant (*P*>0.1). The ATG initiation codon was mutated to ATA in six study subjects and to ATC, ACG, GGG and GTA (one study subject each) in the remainder. In addition, six cases with a mixture of ATG and ATA (one subject), AGG (two subjects) or GTG (three subjects) were not included in the analysis.

### The association of pre-S deletions and BCP double mutations

Because both pre-S deletions and BCP mutations are more prevalent in genotype C than other genotypes (Kao *et al.*, 2003; Sugauchi *et al.*, 2003) it is important to determine whether the BCP mutations are accompanied by pre-S deletions or the reverse. When the samples including those from HCC cases were analysed, no significant difference was found between the BCP mutant group and wild-type group in terms of pre-S deletions (*χ*^2^=0.5974, *P*>0.10; Table 4, top section). As shown above, pre-S deletions are more prevalent in the HCC samples and these samples therefore represent a subset effectively selected for the deletions. For this reason, the control samples were reanalysed without the HCC cases. Again, there is no significant difference in the prevalence of pre-S deletions between the BCP mutant group and wild-type group (*P*=0.5711; Table 4, bottom section), suggesting that the emergence of pre-S deletions and BCP mutations are independent.

### The association of pre-S deletions and genotypes

The effect of genotype on the occurrence of pre-S deletions also was evaluated. In this case–control study, genotypes B and C were found to infect 10.6 % (7/66) and 74.2 % (49/66) of the subjects, respectively. The remainder (15.2 %, 10/66) are infected with a recombinant of genotype C and HBV sequences of unknown genotype (U/C recombinant); details of this recombinant, described originally in Vietnam (which neighbours Guangxi), have been presented previously (Hannoun *et al.*, 2000; Tran *et al.*, 2008). There is no significant difference in the prevalence of pre-S deletions among genotypes B and C and the U/C recombinant, regardless of whether the HCC cases are included in the analysis (Table 5).

## DISCUSSION

This matched nested case–control study reveals that pre-S deletion is an independent risk factor for HCC and its emergence and effect are independent of BCP mutations. The 5′ terminus of pre-S2 is the favoured site for the deletion mutations and the prevalence is significantly higher in HCC than the controls. The prevalence of pre-S deletions is not significantly different between genotypes B and C. The major weakness of this study is that the sample size (HCC cases) is small, so we cannot carry out stratification analysis of a synergistic effect with BCP double mutations in the development of HCC. HBV viral loads have been reported to be associated with the development of HCC and 10^4^–10^5^ copies ml^−1^ was suggested to be the cut-off (Chen *et al.*, 2006b, c). Although we did not measure viral loads for each subject in this study, the influence of viral loads on the development of HCC is comparable between cases and controls; all cases and controls tested positive for HBV DNA with a nested PCR which spans the discontinuity between BCP and the 5′ end of the minus strand of genomic DNA and has a detection limit of around 10^3^–10^5^ genomes ml^−1^ (Fang *et al.*, 2008). The incidence of HCC in HBV cirrhotic patients has been found to be greater than in non-cirrhotic patients (Monto & Wright, 2001). In the Long An cohort, 67 individuals were known to have cirrhosis and 39 of the 61 HCC cases occurred in individuals with cirrhosis (Fang *et al.*, 2008). However, BCP double mutations, per se, are an independent risk factor for the development of liver cirrhosis (Fang *et al.*, 2002; Chen *et al.*, 2005) and cirrhosis was not included in this analysis. HBV-associated HCC may develop in livers with minimal histological changes (Brechot *et al.*, 2000); this is particularly true in populations with a high prevalence of HBsAg, such as in Guangxi, where many adult HBsAg carriers who were infected perinatally may have life-long persistent infections and remain highly immune tolerant with minimal hepatitis, but a high risk of developing HCC. Mutations in enhancer II (C_1653_T) and elsewhere in the basal core promoter (T_1753_V) were found not to be associated with the development of HCC in our study cohort (Fang *et al.*, 2008) and therefore these factors also were not included in this analysis.

Chen *et al.* (2006a) reported that a combination of pre-S deletion mutations and BCP double mutations, rather than either alone, was associated with the development of HCC whilst other case–control studies reported that pre-S deletion is associated with the development of HCC but did not exclude the confounding effect of BCP double mutations (Choi *et al.*, 2007; Lin *et al.*, 2007; Gao *et al.*, 2007). In contrast to the approach using a group case–control study, the matched nested case–control approach used in this study decreases the selection and information bias and increases the comparability. It also proves a causal association of pre-S deletions and HCC because the mutations were detected prior to the development of HCC. Furthermore, our study excludes the confounding effect of BCP double mutations because both case and control have the same type of BCP sequence. Therefore, these results are more reliable than those of previous studies. Recently, Chen *et al.* (2007) reported that pre-S deletions are associated with cirrhosis in HBeAg-negative patients, independent of BCP double mutations.

As noted above, the polymerase ORF is maintained in all isolates regardless of the size and number of deletions. All of the deleted viruses seem to be able to synthesize truncated versions of the large surface protein, with the exception of the isolate from HCC case BO105 which has two extensive deletions and seems unable to make either the large or middle surface protein, although the major surface ORF is intact. It is not clear that such a virus is viable. The sequence from HCC case DH230 is noteworthy; although it is a U/C recombinant (Hannoun *et al.*, 2000), the 33 nt deletion at the beginning of the pre-S1 region resembles that found in genotype D viruses and would permit synthesis of the large surface protein from the second, in-frame methionine codon. The remaining sequences containing deletions, with the exception of two controls (QP244 and QF3), include deletions (often starting at or around nt 3215) that remove all or part of a critical epitope at the beginning of pre-S2. Furthermore, five sequences with deletions (three HCC cases and two control), as well as five sequences without deletions (three HCC cases and two control), have point mutations that destroy the pre-S2 initiation codon. This epitope, 109–123, is HLA class I (A3) and class II (DR2) restricted (Barnaba *et al.*, 1989; Chisari & Ferrari, 1995) and probably the mutations are selected by immune pressure (Fan *et al.*, 2001). However, it is not clear why the mutations are associated with the development of HCC. Hepatocytes expressing modified large (L) and middle (M) surface proteins have a potential growth advantage and may be implicated in the pathogenesis of HBV-related HCC (Fan *et al.*, 2000). The pre-S2 mutant has been reported to upregulate cyclin A expression and induce nodular proliferation of hepatocytes (Wang *et al.*, 2005) and the modified HBsAg may induce oxidative DNA damage and mutations in hepatocytes in the late stages of HBV infection (Hsieh *et al.*, 2004). Furthermore, 3′-truncated pre-S2/S sequences in HBV DNA integrated in HCC have been proposed to enhance tumour development by encoding a protein with transcriptional transactivation activity (Kekule *et al.*, 1990).

Pre-S2 initiation codon mutations may abrogate the expression of M, resulting in pre-S2-defective variants (Raimondo *et al.*, 2004). Such variants have been reported to be associated with advanced liver disease, including HCC (Choi *et al.*, 2007; Wang *et al.*, 2007). In our study, the prevalence of such variants in HCC is higher than in the controls, although the difference is not statistically significant.

It has been reported that the development of pre-S deletion mutations is associated with HBV genotype and their prevalence is higher in genotype C than genotype B in Taiwan, Japan and mainland China (Chen *et al.*, 2006a; Sugauchi *et al.*, 2003; Wang *et al.*, 2007). However, an analysis of samples from 12 countries, including Vietnam, Nepal, Myanmar, China, Korea, Thailand, Japan, Ghana, Russia, Spain, USA and Bolivia, showed that the prevalence of pre-S deletions in genotype C is similar to that in genotype B (25 versus 24.5 %; Huy *et al.*, 2003). In this study, there is no significant difference between genotypes B and C in terms of the prevalence of pre-S deletions. The prevalence of pre-S deletions is higher in the U/C recombinant than genotypes B and C, although the difference is not statistically significant. Recombinant genotypes of HBV have been recognized (Simmonds & Midgley, 2005; Chen *et al.*, 2006a; Wang *et al.*, 2007; Schaefer, 2007) but their epidemiology and association with liver disease need to be investigated further.

In summary, the results of this matched nested case–control study showed that the pre-S deletion mutations, particularly deletions involving the pre-S2 region, are associated with the development of HCC. The pre-S deletion mutations act independently of BCP mutations. The development of pre-S deletions and BCP mutations is independent. There is no significant difference in pre-S deletions between genotypes B and C but the pre-S deletions are more common in the U/C recombinant. However, this is a case–control study and further prospective studies are needed to confirm the role of these mutations in the development of HCC.

## Figures and Tables

**Table 1. t1:** Demographical and clinical data of cases and controls Rows in bold type denote HCC cases with the matched control immediately below.

**Samples**	**Sex**	**Age (years)**	BCP*	**HBeAg**	**Anti-HBe**	**ALT†**	**Genotype**	**Pre-S2 start codon**	Pre-S deletions*
**BC562**	**F**	**31**	**WT**	**−**	**−**	**11**	**C**	**ATG**	**WT**
BB224	F	31	WT	+	−	5	C	ATG	WT
**BL503**	**M**	**52**	**M**	**−**	**+**	**8**	**B**	**ATG**	**WT**
DY008	M	52	M	−	+	7	C	ATG	WT
**BO105**	**M**	**53**	**WT**	**−**	**+**	**34**	**B**	**Deleted**	**2606–2876, 2998–25**
NC073	M	53	WT	−	+	39	C	ATA	WT
**BX72**	**F**	**30**	**M**	**−**	**+**	**8**	**C**	**Deleted**	**3205–2**
CB403	F	30	M	−	+	17	C	ATA	3042–3077, 3213–54
**CZ662**	**M**	**44**	**M**				**C**	**ATG**	**WT**
BL137	M	44	M	−	+	5	C	ATG	WT
**DH230**	**F**	**46**	**WT**				**U/C**	**ATG**	**2849–2880**
DL370	F	46	WT				B	Deleted	3152–35
**DM207**	**M**	**36**	**M**	**−**	**+**	**10**	**B**	**ATG**	**WT**
GG311	M	36	M	−	+	7	C	ATG	WT
**GY177**	**M**	**35**	**M**	**−**	**+**	**96**	**C**	**ATC**	**WT**
JD90	M	35	M	−	+	37	C	ATG	WT
**JC17**	**M**	**42**	**M**				**C**	**ATA**	**WT**
GA130	M	42	M	−	+	140	U/C	ATG	WT
**JO46**	**F**	**36**	**M**				**C**	**ATG**	**3215–56**
JS660	F	36	M	−	+	53	C	ATG	WT
**JS518**	**M**	**50**	**M**	**−**	**+**	**73**	**U/C**	**ATG**	**3209–47**
QP208	M	51	M	−	+	5	B	ATG	WT
**NA102**	**M**	**54**	**M**	**−**	**+**	**5**	**C**	**ATG**	**WT**
GM240	M	54	M	−	+	53	B	ATG	WT
**NA407**	**M**	**46**	**M**				**U/C**	**ATG**	**3215–53**
GG091	M	46	M	−	+	7	C	ATG	WT
**DB272**	**M**	**48**	**M**	**−**	**+**	**27**	**B**	**ATG**	**WT**
NN079	M	50	M	−	+	55	C	ATG	WT
**ND10**	**M**	**39**	**M**				**C**	**Deleted**	**3141–51**
NS133	M	39	M				C	Deleted	3151–55
**NS052**	**F**	**38**	**M**	**−**	**+**	**37**	**U/C**	**ATG**	**3215–5**
QP244	F	38	M	−	+	10	U/C	ATG	2888–3082
**PL010**	**M**	**37**	**M**	**−**	**+**	**34**	**C**	**ATG**	**3215–50**
NX083	M	37	M				C	ATG	WT
**QB002**	**M**	**40**	**M**	**−**	**+**	**84**	**C**	**ATG**	**WT**
NW204	M	40	M	−	+	7	C	ATG	WT
**QD208**	**M**	**48**	**M**	**−**	**+**	**5**	**C**	**GGG**	**WT**
QZ034	M	45	M	−	+	90	C	ATG	WT
**QG211**	**M**	**41**	**M**	**−**	**+**	**15**	**C**	**ATA**	**2981–3083, 3215–5**
QF3	M	41	M	−	+	17	C	ATG	3004–3183
**QL367**	**M**	**35**	**M**	**−**	**−**	**37**	**C**	**ATG**	**WT**
PP016	M	35	M	−	+	8	C	ATG	WT
**QP046**	**M**	**34**	**M**				**U/C**	**ATG**	**WT**
QG364	M	34	M	−	+	7	C	ATG	WT
**QP257**	**M**	**45**	**M**	**−**	**+**	**24**	**C**	**ATA**	**3215–51**
QS582	M	45	M	−	+	5	C	ATG	WT
**QW026**	**F**	**52**	**M**	**−**	**−**	**5**	**C**	**ATG**	**WT**
BH3	F	53	M	**−**	+	37	C	ATG	WT
**QW037**	**M**	**48**	**M**	−	**+**	**31**	**C**	**ATG**	**34–54**
QQB73	M	48	M	**−**	+	31	C	ATG	WT
**TD019**	**M**	**38**	**M**	**−**	**+**	**25**	**C**	**ATG**	**WT**
QL523	M	38	M	−	+	34	U/C	ATA	WT
**TX164**	**M**	**40**	**M**	**−**	**+**	**44**	**U/C**	**ATG**	**WT**
TZ027	M	40	M	**−**	+	27	C	ATG	WT
**WX116**	**M**	**52**	**M**	−	**+**	**18**	**C**	**ATG**	**WT**
BH419	M	52	M	**−**	+	31	C	ACG	3215–53
**XA282**	**M**	**45**	**M**	**−**	**+**	**48**	**C**	**Deleted**	**3132–54**
XW217	M	45	M	−	+	0	C	ATG	WT
**XD241**	**M**	**42**	**WT**	**−**	**+**	**12**	**U/C**	**ATG**	**3215–57**
XW73	M	42	WT	+	−	22	C	ATG	WT
**XW230**	**F**	**50**	**M**				**C**	**ATG**	**WT**
YF336	F	50	M	**−**	−	12	C	ATG	WT
**YJ010**	**F**	**47**	**M**	−	**+**	**5**	**C**	**ATG**	**WT**
QF100	F	47	M	**−**	+	3	C	ATG	WT
**YN409**	**M**	**55**	**M**	**−**	**+**	**5**	**C**	**GTA**	**3215–5**
YF003	M	55	M	−	+	35	C	ATG	WT

*WT, Wild type; M, mutant. †Cut-off ≥40 IU.

**Table 2. t2:** Pre-S deletions and HCC Pre-S deletions between cases and control: McNemar's test *χ*^2^=7.3636, *P*<0.01. Pre-S deletions between males and females: Pearson's *χ*^2^ test *χ*^2^=1.386, *P*>0.10.

**Groups**	**All samples**			**Male samples**			**Female samples**		
	**No.**	**Pre-S deletion**	**Deletion rate (%)**	**No.**	**Pre-S deletion**	**Deletion rate (%)**	**No.**	**Pre-S deletion**	**Deletion rate (%)**
HCC	33	15	45.5	25	11	44.0	8	4	50.0
Control	33	6	18.2	25	3	12.0	8	3	37.5
Total	66	21	31.8	50	14	28.0	16	7	43.8

**Table 3. t3:** Univariate and multivariate analysis for factors associated with development of HCC

**Analysis**	**Variable**	**Parameter estimate**	**Standard error**	***χ*^2^**	**Proportion >*χ*^2^**	**Hazard ratio**	**Hazard ratio 95 % confidence limits**	
Univariate analysis	HBeAg	−17.20289	3846	0.0000	0.9964	0.000	0.000	
	Anti-HBe	−0.69303	1.22472	0.3202	0.5715	0.500	0.045	5.514
	ALT	0.22314	0.67082	0.1107	0.7394	1.250	0.336	4.655
	Genotype	0.32187	0.47212	0.4648	0.4954	1.380	0.547	3.481
	Pre-S	2.30256	1.04880	4.8199	0.0281	10.000	1.280	78.114
Multivariate analysis	Pre-S	1.94591	1.06904	3.3132	0.0687	7.000	0.861	56.894

**Table 4. t4:** Pre-S deletions and HBV core promoter mutations Samples including HCC (Pearson's *χ*^2^ test) *χ*^2^=0.5974, *P*>0.10. Control samples (McNemar's test) *P*=0.5711.

	**Core promoter**	**No. samples**	**Pre-S deletion**	**Deletion rate (%)**
Samples including HCC	Mutations (A_1762_T, G_1764_A)	58	17	29.3
	Wild type	8	4	50.0
Samples without HCC	Mutations (A_1762_T, G_1764_A)	29	5	17.2
	Wild type	4	1	25.0

**Table 5. t5:** Pre-S deletions and HBV genotype Pearson's *χ*^2^ test *χ*^2^=4.3265, *P*>0.10.

**Genotypes**	**All samples**		
	**No. samples**	**Pre-S deletion**	**Deletion rate (%)**
Genotype B	7	2	28.6
Genotype C	49	13	26.5
Genotype U/C	10	6	60.0
Total	66	21	31.8
